# Impaired phagocytosis and reactive oxygen species production in phagocytes is associated with systemic vasculitis

**DOI:** 10.1186/s13075-016-0994-1

**Published:** 2016-04-22

**Authors:** Åsa CM Johansson, Sophie Ohlsson, Åsa Pettersson, Anders A. Bengtsson, Daina Selga, Markus Hansson, Thomas Hellmark

**Affiliations:** Department of Haematology, Lund University and Skåne University Hospital, BMC B13, 221 84 Lund, Sweden; University and Regional Laboratories Region Skåne, Clinical Immunology and Transfusion Medicine, Skåne, 221 85 Lund, Sweden; Department of Clinical Sciences Lund, Nephrology, Lund University, Skane University Hospital, Lund, Sweden; Department of Clinical Sciences, Lund, Rheumatology, Lund University, Skåne University Hospital, Lund, Sweden

**Keywords:** ANCA, Vasculitis, PMN, Monocytes, Oxidative burst, Phagocytosis

## Abstract

**Background:**

Anti-neutrophil cytoplasmic antibodies associated vasculitides (AAV) is a group of autoimmune diseases, characterized by small vessel inflammation. Phagocytes such as neutrophils and monocytes are the main effector cells found around the inflamed vessel wall. Therefore, we wanted to investigate aspects of function and activation of these cells in patients with AAV.

**Methods:**

Flow cytometry was used to evaluate: the expression of activation markers (CD11c, CD62L, CD177 and C5aR); the number of recently released neutrophils from bone marrow, defined as CD10^-^D16^low^ cells in peripheral blood; and the capacity of peripheral blood monocytes and polymorphonuclear leukocytes (PMN) to produce reactive oxygen species and to phagocytose opsonized bacteria.

**Results:**

AAV patients (n = 104) showed an increase of CD10^-^CD16^low^ neutrophils and total PMN in peripheral blood, suggesting a combination of increased bone marrow release and prolonged survival. An increased percentage of AAV PMN expressed CD177 but no other signs of activation were seen. A decreased production of reactive oxygen species was observed in AAV phagocytes, which was associated with disease activity. Moreover, granulocytes from patients with microscopic polyangiitis showed lower oxidative burst capacity compared to patients with granulomatosis with polyangiitis or eosinophilic granulomatosis with polyangiitis. In addition, decreased phagocytosis capacity was seen in PMN and monocytes.

**Conclusion:**

Our results indicate that phagocytes from AAV patients have impaired function, are easily mobilized from bone marrow but are not particularly activated. The association between low reactive oxygen species formation in PMN and disease severity is consistent with findings in other autoimmune diseases and might be considered as a risk factor.

**Electronic supplementary material:**

The online version of this article (doi:10.1186/s13075-016-0994-1) contains supplementary material, which is available to authorized users.

## Background

Anti-neutrophil cytoplasmic antibody associated vasculitides (AAV) is a group of autoimmune diseases comprising eosinophilic granulomatosis with polyangiitis (EGPA), granulomatosis with polyangiitis (GPA) and microscopic polyangiitis (MPA). AAV are conditions defined by autoimmune small vessel inflammation [1] and characterized by autoantibodies, the anti-neutrophil cytoplasmic antibodies (ANCA). The ANCA antigens proteinase 3 (PR3) and myeloperoxidase (MPO) are mainly present in the primary granules of neutrophils and in the peroxidase positive lysosomes of monocytes.

Neutrophils are found in abundance in and around inflamed vessel walls in patients with AAV, and ANCA bound to the target antigens leads to activation of neutrophil effector functions and ultimately tissue damage [2]. In vitro experiments show that ANCA-mediated activation of primed neutrophils leads to degranulation and production of reactive oxygen species (ROS), complement activation [3], and release of neutrophil extracellular traps (NETs) [4]. Furthermore, the pathological role of ANCA has been shown in animal models where ANCA cause vasculitis-like symptoms [5, 6].

Monocytes have been less studied than neutrophils, but are known to be prominent in AAV lesions [7]. There are reports showing that ANCA can activate monocytes and early exudative macrophages but not mature macrophages [8–10].

Monocytes and PMN, such as neutrophils, are produced in the bone marrow and released into the circulation. Inflammation causes increased mobilization of PMN from the bone marrow that is thought to be reflected by an increased percentage of CD10^-^CD16^low^ neutrophils in peripheral blood [11, 12]. Upon activation the expression of various surface proteins changes, e.g., C5aR and CD62L are downregulated [13, 14] whereas increased CD11c expression is observed [15]. In addition to the changing expression of their surface proteins, activated monocytes and neutrophils are primed to release granules and produce ROS by the nicotinamide adenine dinucleotide phosphate-oxidase (NADPH) complex [16]. ROS are major effector molecules in inflammatory processes and tightly linked to NET formation. During the last decade, an increasing amount of data have been produced to support a T cell regulating role for monocyte-produced and neutrophil-produced ROS [17–20].

This study aimed to characterize monocytes and PMN from patients with AAV, in regard to function, bone marrow release and activation, in order to understand the role of these phagocytes in AAV and autoimmunity.

## Methods

### Patients and controls

Patients with AAV were recruited to the study when attending their scheduled visit at the Department of Nephrology or Rheumatology, Skåne University Hospital, Lund, Sweden. Classification of AAV was done according to the consensus methodology described by Watts et121 al. in 2007 [21]. Patients with AAV (n = 104) included in the study comprised 73 patients with GPA, median age 66 years (range 20–84 years), 23 patients with MPA, median age 72 years (range 20–84 years), and 8 patients with EGPA, median age 71 years (range 56–78 years). Patients with EGPA in this study had normal eosinophil counts at the time of sampling, with a mean of 0.2 × 10^9^/L, range 0.01–0.6 × 10^9^/L (reference range <0.7 × 10^9^/L). Disease activity was assessed using the Birmingham Vasculitis Activity Score version 3 (BVAS 3) [22]. Demographic and clinical characteristics are shown in Table 1. ANCA specificity was determined with ELISA at an accredited clinical laboratory (Wieslab AB, Malmö, Sweden).

**Table 1:** Patients characteristics and demographics

	All patients (n = 104)	Remission BVAS3 = 0 (n = 82)	Disease activity BVAS3 > 1 (n = 22)
Age, years, median (range)	68 (20–86)	68 (25–84)	66 (20–86)
Disease duration, years, median (range)	7 (0–50)	7 (0–50)	1 (0–31)
GPA, % (*n*)	70 % (n = 73)	68 % (n = 56)	78 % (n = 18)
MPA, % (*n*)	22 % (n = 23)	24 % (n = 19)	17 % (n = 4)
EGPA, % (*n*)	8 % (n = 8)	8 % (n = 7)	4 % (n = 1)
MPO-ANCA, % (*n*)	30 % (n = 32)	33 % (n = 26)	26 % (n = 6)
PR3-ANCA, % (*n*)	59 % (n = 61)	56 % (n = 46)	70 % (n = 16)
BVAS3, median (range)	0 (0–16)	0	4 (1–16)
Leukocytes, 10^9^/L, median (range)^a^	6.4 (3.0–13.7)	6 (3.2–13.7)	7.9 (3.0–12)
P-creatinine, μmol/L, median (range)	98 (54–646)	96 (59–635)	143 (54–646)
P-CRP, mg/L, median (range)	2.6 (<0.6–92)	2.6 (<0.6–27)	5.7 (<0.6–92)
*Treatment* ^b^			
Prednisone, % (median dose of treated patients)	60 % (6.25 mg)	55 % (5 mg)	77 % (15 mg)
Azathioprine, % (*n*)	28 % (n = 29)	31 % (n = 26)	13 % (n = 3)
Mycophenolate mofetil, % (*n*)	8 % (n = 8)	10 % (n = 8)	0
Rituximab, % (*n*)	19 % (n = 20)	19 % (n = 17)	17 % (n = 3)
Methotrexate, % (*n*)	14 % (n = 15)	14 % (n = 11)	17 % (n = 4)
Cyclophosphamide, % (*n*)	11 % (n = 11)	7 % (n = 6)	27 % (n = 6)

*BVAS* Birmingham Vasculitis Activity Score version 3, *GPA* granulomatosis with polyangiitis, *MPA* microscopic polyangiitis, *EGPA* eosinophilic granulomatosis with polyangiitis, *MPO* myeloperoxidase, *ANCA* anti-neutrophil cytoplasmic antibodies, *PR3* proteinase 3, *CRP* C-reactive protein. ^a^Reference range 3.5–8.8 10^9^/L. ^b^There were 14 patients who did not receive any treatment

As disease controls, patients (n = 26) with systemic lupus erythematosus (SLE) were recruited to the study, when attending their scheduled visit at the Department of Rheumatology, Skåne University Hospital, Lund, Sweden. All patients fulfilled at least four American College of Rheumatology classification criteria for SLE [23]. Disease activity was assessed using the Systemic Lupus Erythematosus Disease Activity Index 2000 (SLEDAI-2 K) [24], and organ damage was evaluated according to the Systemic Lupus International Collaborative Clinics/American College of Rheumatology damage index (SLICC/ACR-DI) [25]. Blood donors (Blood center in Lund) and healthy volunteers were recruited as controls (n = 112). Demographic data on age and gender were available for 102 of the 112 controls, giving a median age of 49 years (range 19–74) and female to male ratio of 1.1 to 1.0. Patients with AAV and controls were analyzed in parallel between 2011 and 2014. Patients with SLE were analyzed in parallel with healthy controls and Patients with AAV during 2014. The Regional Ethics Board in Lund, Sweden (LU) approved the study and informed consent was obtained from all participants.

### Phagocytosis and oxidative burst

Peripheral blood from patients and controls was collected in vacutainer tubes containing sodium heparin (Becton Dickinson, BD, New York, NY, USA). All samples were analyzed within 24 h. The samples were stored at room temperature and protected from light until analyzed. Phagocytosis was investigated using the in vitro diagnostic (IVD)-labeled PhagoTest assay (Glycotope Biotechnology, GmBH, Germany), according to the manufacturer’s protocol. This flow-cytometry-based method measures the percentage of PMN and monocytes having ingested fluorescein-labeled opsonized *Escherichia coli* (*E. coli*), and mean fluorescence intensity (MFI) corresponds to the number of ingested bacteria per cell. Production of ROS in peripheral blood PMN and monocytes was investigated using the IVD-labeled PhagoBurst assay, (Glycotope Biotechnology, GmBH, Germany), according to the manufacturer’s protocol, after ex vivo activation with phorbol-12-myristate-13-acetate (PMA) or opsonized *E. coli*. At least 15,000 PMN were collected based on forward and side scatter properties. No patient had ROS deficiency.

### Phagocyte phenotypes

Peripheral blood from patients and controls was collected in vacutainer tubes containing sodium heparin (Becton Dickinson, BD, New York, USA). All sample were analyzed within 24 h. The samples were stored at room temperature and protected from light until analyzed. The expression of selected surface markers on phagocytes was analyzed using flow cytometry. Briefly, peripheral blood was lysed, using 0.84 % ammonium chloride. The remaining leukocytes were stained for surface expression of CD10, CD16, CD62L (BD Bioscience, San Jose, CA, USA), C5aR (CD88), CD11c (Biolegend, San Diego, CA, USA) and CD177 (AbD Serotec, Raleigh, NC, USA) and finally analyzed using a FACSCanto II and the DIVA software (Becton Dickinson, Franklin Lakes, NJ, USA). Gating strategies are shown in Additional file 1: Figure S1.

### Statistical analysis

Correlation was assessed by Spearman’s test. The Mann-Whitney *U* test was used for two-group comparisons, and the Kruskal-Wallis and Dunn multiple comparisons test was for three or more groups. All *p* values were considered significant at *p* < 0.05. Prism for MacOS version 5.0a was used for statistic calculations.

## Results

### Patient characteristics

The clinical and demographic characteristics of the patients with AAV (n = 104) at the time of sampling are reported in Table 1. The majority of the patients were diagnosed with GPA (70 %), whereas patients with MPA and EGPA represented 22 % and 8 % of the cohort, respectively. Most patients were in remission and a little over 20 % of the patients had some kind of disease activity according to the BVAS3, with a median score of 4 (range 1–16). The median age of the patients with SLE was 62 years (range 25–85) and the female-to-male ratio was 3.5 to 1.0. Most SLE patients were in remission or had low disease activity with a mean SLEDAI-2 K of 2 (range 0–6). The disease severity, defined as organ damage, was evaluated using SLICC with a median score of 2 (range 0–6). Of the SLE patients, 60 % were treated with prednisone (the mean dose in treated patients was 5 mg/day, range 2.5–20.0 mg/day); 64 % were treated with hydroxychloroquine and 54 % were treated with other immune modulating drugs (methotrexate, mycophenolate mofetil or azathioprine).

### Signs of increased mobilization of PMN from bone marrow in patients with AAV

The frequency of phagocytes and lymphocytes was evaluated in peripheral blood from patients with AAV (n = 104) and healthy controls (n = 112). Patients had an increased percentage of PMN and a decreased number of lymphocytes compared with controls (*p* < 0.0001). There was no difference in the number of monocytes (Table 2).

**Table 2:** Monocyte and PMN phenotypes

Phenotype	Patients with AAV	Healthy controls	*P* value
Monocytes (% of leukocytes)	4.4 ± 0.3	4.8 ± 0.24	*n.s.*
(10^9^/L, reference range <1,1)	0.33 ± 0.02		
Lymphocytes (% of leukocytes)	20 ± 1.2	31 ± 1.2	*<0.0001*
(10^9^/L, reference range 1.1–4.8)	0.74 ± 0.05		
PMN (% of leukocytes)	39 ± 1.3	28 ± 1.1	*<0.0001*
(10^9^/L, reference range 1.7–8.0)	5.9 ± 0.3		
CD16^+^CD10^+^ (% of PMN)	84 ± 1.1	82 ± 1.2	*n.s.*
CD16^dim^CD10^-^ (% of PMN)	8.9 ± 0.8	6.8 ± 0.6	*0.0109*
CD177^+^ (% of PMN)	55 ± 2.4	47 ± 1.9	*0.0075*
CD10^+^CD16^+^ PMN			
CD88^+^ (geoMFI)	442 ± 14	409 ± 15	*n.s*
CD62L^+^ (geoMFI)	1,277 ± 37	1,269 ± 41	*n.s.*
CD11c^+^(geoMFI)	558 ± 14	596 ± 16	*n.s*
CD10^+^CD16^dim^ PMN			
CD88^+^ (geoMFI)	391 ± 8.2	381 ± 7.6	*n.s*
CD62L^+^ (geoMFI)	450 ± 23	457 ± 22	*n.s*
CD11c^+^(geoMFI)	354 ± 7.2	428 ± 10	*<0.0001*
Monocytes			
CD88^+^ (geoMFI)	162 ± 2.5	165 ± 2.5	*n.s*
CD62L^+^ (geoMFI)	770 ± 29	651 ± 37	*<0.0001*
CD11c^+^(geoMFI)	1,645 ± 63	2,079 ± 78	*<0.0001*

The frequencies of monocytes, lymphocytes, polymorphonuclear leukocytes (PMN), CD10^+^CD16^+^ (mainly segment nucleated neutrophils) and CD10^-^CD16^dim^ (suggested as a marker for newly released neutrophils) were investigated in healthy controls (n = 109) and patients (n = 105) using flow cytometry. In addition, the level of surface expression of CD88 (C5aR), CD62L and CD11c was studied on CD10^+^ CD16^+^ PMN, CD10 ^+^ CD16^dim^ PMN and monocyte populations, as a measurement of activation and reported as geometric mean fluorescence intensity (geoMFI). The two-sided Mann-Whitney test was used to calculate the level of significance. Values are reported as mean ± SEM. *AAV* anti-neutrophil cytoplasmic antibodies associated vasculitides, *n.s.* not significant

Elevated numbers of CD10^-^CD16^low^ PMN in peripheral blood are thought to reflect increased mobilization of neutrophils from the bone marrow [11, 12]. Patients with AAV had a greater percentage of CD10^-^CD16^low^ PMN (*p* = 0,0079) (Table 2), suggesting elevated bone marrow release. There was no difference in the percentage of mature segment nucleated (CD10^+^CD16^+^) neutrophils.

In neutrophils, surface translocation of proteinase 3 (PR3), one of the main ANCA targets, appears to occur in association with CD177 [26–28]. In AAV-PMN there were increased levels of CD177 on neutrophils (*p* = 0.0061) (Table 2), which could indicate increased delivery of PR3 to the cell surface of neutrophils. No CD177 was observed on monocytes (data not shown).

To characterize the activation status of phagocytes in peripheral blood, the expression of CD88 (C5aR), CD11c, and CD62L were investigated. Mature segment nucleated neutrophils (CD10^+^CD16^+^) from patients with AAV had similar surface expression of these molecules as controls (Table 2). Less surface expression of CD11c was observed on the CD10^+^CD16^dim^ PMN and monocytes from patients with AAV. AAV monocytes also had increased expression of CD62L (Table 2). Thus, phagocytes in patients with AAV were not more activated than in healthy controls.

### Decreased production of ROS in AAV phagocytes

**Fig. 1:**
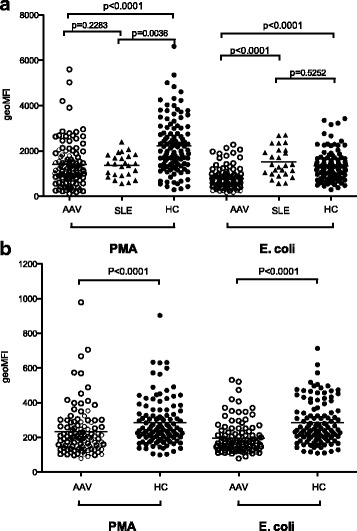
Phagocytes from patients with anti-neutrophil cytoplasmic antibodies associated vasculitides (*AAV*) produced fewer reactive oxygen species (ROS) than phagocytes from healthy blood donors. The capacities of polymorphonuclear leukocytes (*PMN*) (**a**) or monocytes (**b**) from healthy controls (*HC*) (n = 112), patients with AAV (n = 104), and patients with systemic lupus erythematosus (SLE) (n = 26) (only PMN) to produce ROS upon activation with phorbol 12-myristate 13-acetate (*PMA*) or opsonized *E. coli* were investigated using flow cytometry. The amount of ROS produced is shown as geometric mean fluorescence intensity (*geoMFI*). The two-sided Mann-Whitney test was used to calculate the level of significance. *Horizontal lines* represent the median value of each dataset

ROS production is an important effector function of phagocytes for defense against microbes, but also in the regulation of the innate and adaptive immune system [19, 29]. Production of ROS in phagocytes is generated by the NADPH oxidase that resides in the plasma membrane (5 %) and in granule membranes (95 %) in neutrophils. To evaluate phagocyte function in AAV, we decided to investigate intracellular ROS production. PMN and monocytes in peripheral whole blood from patients with AAV (n = 104) (Table 1), SLE (n = 26), and healthy controls (n = 112), were stimulated with either the protein kinase C activator, PMA, or with opsonized *E. coli*. AAV phagocytes, both PMN and monocytes, showed decreased capacity to produce ROS after activation with phorbol 12-myristate 13-acetate (PMA) (*p* < 0.0001) or *E. coli* (*p* < 0.0001, Fig. 1a and b) compared with healthy controls.

PMN from patients with AAV had less ROS formation compared with PMN from patients with SLE after *E. coli* stimulation (*p* < 0.0001) (Fig. 1a), but no significant difference was observed after PMA activation.

**Fig. 2:**
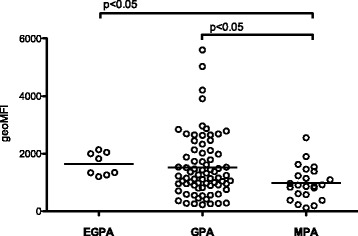
Patients with microscopic polyangiitis (*MPA*) had lower reactive oxygen species (ROS) formation compared with patients with granulomatosis with polyangiitis (*GP*A) or eosinophilic granulomatosis with polyangiitis (*EGPA*). ROS production was measured by flow cytometry after ex vivo activation of peripheral blood PMN with phorbol 12-myristate 13-acetate. Patients with anti-neutrophil cytoplasmic antibodies associated vasculitides were divided based on disease characteristics into EGPA, GPA or MPA. The amount of ROS produced is shown as geometric mean fluorescence intensity (*geoMFI*). The Kruskal-Wallis test with Dunn’s multiple comparison test was used to calculate the level of significance. *Line* represents the median value of each dataset

The patients were divided based on disease characteristics into EGPA, GPA, or MPA. PMN from patients with MPA (n = 23) had decreased burst capacity compared to GPA (n = 73; *p* < 0.05) and EGPA patients (n = 8; *p* < 0.05) after PMA activation (Fig. 2), whereas no significant differences were observed after *E. coli* stimulation (data not shown). The ROS producing capacity was not associated with the presence of PR3-ANCA or MPO-ANCA (data not shown).

### Decreased ROS formation is associated with disease activity

**Fig. 3:**
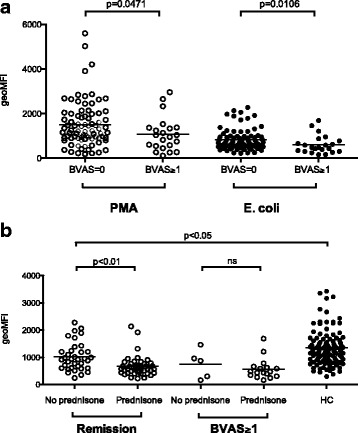
Disease activity in patients with anti-neutrophil cytoplasmic antibodies associated vasculitides (AAV) is associated with decreased production of reactive oxygen species (ROS). ROS production was measured by flow cytometry after ex vivo activation of peripheral blood polymorphonuclear leukocytes (PMN) with phorbol 12-myristate 13-acetate (*PMA*) or opsonized *E. coli* in **a** patients with AAV in remission (Birmingham Vasculitis Activity Score version 3 (*BVAS*) = 0) or with some kind of disease activity (BVAS ≥1). **b** Patients are additionally divided based on prednisone treatment or not. The amount of ROS produced is shown as geometric mean fluorescence intensity (*geoMFI*). The two-sided Mann-Whitney test was used to calculate the level of significance between two groups and the Kruskal-Wallis test with Dunn’s multiple comparison test was used to calculate the level of significance between more than two groups. *Line* represents the median value of each dataset. *HC* healthy controls

The severity of autoimmune diseases has previously been associated with decreased ROS production [30–32]. Hence, to study if the disease activity of AAV was associated with changes in ROS production, the patients were divided in two groups based on disease activity according to the BVAS3 (Table 1). PMN from patients with BVAS3 ≥ 1 had decreased ROS production, compared with patients in remission, when activated with *E. coli* (*p* = 0,0306) (Fig. 3a) and PMA (*p* = 0,0471). Most patients in the disease activity group were diagnosed as having GPA (n = 17) (Table 1). There was no association between disease activity and ROS production in monocytes (data not shown).

Prednisone treatment is common in this patient group and affects phagocyte function. In this study, 60 % of the patients were on prednisone treatment, with a mean dose of 7.5 mg (range 2–80 mg) (Table 1). To investigate the corticosteroid effect on ROS production, the patients with disease activity (BVAS3 ≥ 1) or in remission (BVAS3 = 0) were divided based on prednisone treatment or not (Fig. 3b). Patients in remission without prednisone had a higher burst capacity compared with patients on prednisone treatment after *E. coli* stimulation (*p* < 0.01). However, patients in remission who were not receiving prednisone treatment still had a decreased burst compared to healthy controls (*p* < 0.05). In relation to corticosteroid treatment, there was no difference in burst capacity in patients with BVAS ≥1. Similar results were seen after PMA activation. One patient in the group with disease activity was newly diagnosed and not on any treatment at the time of sampling. PMN from this patient had decreased burst activity (geometric mean fluorescence intensity of 875 and 1053 after *E. coli* or PMA activation, respectively). In line with the PMN, the monocytes from this patient also produced decreased amounts of ROS (171 and 218 after *E. coli* or PMA activation, respectively (Fig. 3b).

### Decreased phagocytosis in patients with AAV

**Fig. 4:**
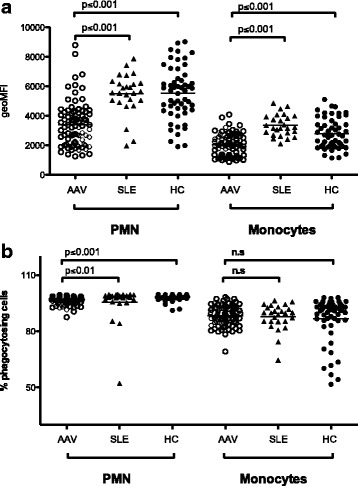
Decreased phagocytosis in anti-neutrophil cytoplasmic antibodies associated vasculitides (*AAV*). To evaluate further the function of phagocytes in AAV, the capacity to phagocytose opsonized *E. coli* was investigated in polymorphonuclear leukocytes (*PM*N) and monocytes from patients with AAV (n = 84), patients with systemic lupus erythematosus (n = 26), and healthy controls (*HC*) (n = 54). **a** Amount of phagocytosed *E. coli* bacteria shown as geometric mean fluorescence intensity (*geoMFI*). **b** Percentage of phagocytosing cells. Differences between healthy controls, patients with SLE, and patients with AAV were calculated using the Kruskal-Wallis test with Dunn’s multiple comparison test, and the following *p* values were obtained for all three groups: geoMFI of PMN, *p* < 0.0001; geoMFI of monocytes, *p* < 0.0001 and % phagocytosing cells of PMN, *p* < 0.0001; and % phagocytosing cells of monocytes, *p* = 0.4091. *P* values presented in the figure are for comparison between two groups

Another important function of phagocytes is the phagocytosis of antibody-coated microbes and foreign material, which often precedes the intracellular ROS production. The capacity to phagocytose opsonized *E. coli* was investigated in PMN and monocytes from patients with AAV (n = 83) or SLE (n = 26) and controls (n = 54). Both AAV PMN and monocytes had decreased phagocytosis capacity, defined as the amount of phagocytosed *E. coli* per cell compared with patients with SLE and healthy controls (*p* < 0.0001) (Fig. 4a). The AAV PMN also had a decreased percentage of phagocytosing cells (*p* < 0.0001) (Fig. 4b). There was no association with disease activity, corticosteroids or ANCA specificity (data not shown).

## Discussion

PMN and monocytes from patients with AAV were characterized with respect to bone marrow release, activation and function. This study shows that patients with AAV have increased mobilisation of neutrophils from the bone marrow, impaired phagocyte functions, such as ROS production and phagocytosis, but normal levels of most phagocyte activation markers.

In line with earlier observations, we found that patients with AAV had a decreased percentage of lymphocytes and an increase of PMN. Low levels of lymphocytes are common on diagnosis of AAV and during flares [33] and might also be an effect of immune suppressive treatment [34, 35]. The increased percentage of PMN in patients with AAV could be explained by increased survival in the periphery in combination with elevated bone marrow release. We have shown previously that AAV neutrophils have a lower rate of apoptosis and longer in vitro survival compared with healthy controls and patients with SLE [36]. Furthermore, patients with AAV appeared to have an increased release of neutrophils from the bone marrow, measured as elevated numbers of CD16^dim^CD10^-^ neutrophils in peripheral blood. Increased levels of CD177 were observed on PMN, but there were no other obvious signs of activation. The elevated expression of CD177 on PMN is in concordance with prior findings [26, 27].

Our data support that patients with AAV have a decreased capacity to produce ROS in vivo. The association with disease activity further strengthened our finding. Low ROS production has been associated with disease severity in other autoimmune conditions, including Behcet’s disease [30], Guillian-Barre syndrome [31], multiple sclerosis [32] and SLE [37], and might be a common denominator in maintaining chronic inflammation. Moreover, recent findings in an ANCA-induced glomerulonephritis model using mice deficient in either of the NADPH oxidase subunits, gp91^phox^ or p47^phox^ [38], suggested that ROS limit ANCA-induced inflammation by downregulating caspase 1 and hence keep the inflammasome in check. This finding supports an important role of ROS in regulating vasculitis.

In patients with AAV, monocytes and PMN had decreased capacity to phagocytose compared to patients with SLE. This effect could not be explained by differences in age or treatment between the patient cohorts. Furthermore, patients with AAV tended towards more compromised burst formation than patients with SLE. Using, the same methods as in this report, Taylor et al. saw a similar scenario in which patients with subacute liver failure had decreased phagocytosis, whereas patients with acute liver failure or sepsis did not differ from healthy controls [39]. Future studies are needed to explain these differences.

Patients with MPA had decreased ROS production compared to patients with GPA or EGPA, suggesting more impaired phagocyte function in these patients. The patients with MPA were slightly older than those with GPA (median age 72 and 66 years, respectively) and an age-dependent difference in the effect on ROS production in these two groups could not be excluded; however, no correlation between age and ROS production was seen (data not shown). Future studies designed to investigate this difference would be of interest.

Observations from a genome-wide association study have suggested that PR3-AAV and MPO-AAV are distinct autoimmune syndromes with different genetic phenotypes [40]. Interestingly, by dividing patients based on ANCA specificity, rather than clinical diagnosis, no differences in ROS production were observed. This indicates that decreased ROS formation might be associated with chronic inflammatory processes giving rise to the clinical picture, rather than specifically linked to ANCA pathogenesis.

Corticosteroids have been reported to affect ROS production in PMN in a cumulative dose-dependent way [41], and it is presently unclear whether this effect is due to increased disease severity. In a previous report on PMN function in SLE there was no correlation between corticosteroid dose and the amount of intracellular ROS produced. However, the patients were receiving relatively low doses of corticosteroids (mean = 5 mg oral prednisone/day in treated patients) [37]. In the present study we observed that patients in remission who were receiving ongoing prednisone treatment had decreased burst compared with patients not on prednisone, suggesting that in this case a minor effect of prednisone on ROS formation could not be excluded. On the other hand, patients with a tendency to recurrent flares or having low grade activity (not captured by the BVAS) frequently remain on prednisone treatment during remissions, hence patients in remission who are on prednisone treatment might have another kind of inflammatory phenotype that is reflected by decreased ROS formation.

In general, prednisone treatment could not explain the decreased ROS production in patients with AAV, as the patients in remission who were not being treated with prednisone had a decreased burst compared with controls. In addition, a newly diagnosed patient who was not on any treatment at the time of sampling had low burst activity.

We have previously reported that PR3-ANCA induced increased intracellular but not extracellular ROS production in PMN from patients with AAV [42]. The activation and control of the NADPH oxidase in neutrophils (NOX2) is incompletely understood. Different agonists encountered by the neutrophil engage different combinations of kinases, and thereby affect the degree of activity of the NADPH complex, and in the end the amount of ROS produced [43]. Hence, the different results in PR3-ANCA-induced ROS on one hand and *E. coli* or PMA on the other hand, might be due to the different routes of NADPH activation: PR3-ANCA activates by binding to membrane-bound PR3; opsonized *E. Coli* by receptor mediated phagocytosis; and PMA by activation of protein kinase C.

## Conclusions

The phagocytes in AAV were found to have decreased functions such as lower ROS formation and phagocytosis, compared to healthy controls. The decreased ROS production is consistent with findings in other autoimmune diseases and might be a risk factor in autoimmunity and a common denominator maintaining chronic inflammation. Although increased expression of CD177 was observed on PMN, no other clear signs of activation were seen. The increased percentage of PMN in peripheral blood might be due to a combination of increased bone marrow release and prolonged survival. Future studies will illuminate the role of ROS formation and PMN in AAV and autoimmunity.

## Additional file

Additional file 1: Gating strategies. (DOCX 188 kb)

## Abbreviations

AAV: anti-neutrophil cytoplasmic antibodies associated vasculitides
ANCA: the anti-neutrophil cytoplasmic antibodies
BVAS3: Birmingham Vasculitis Activity Score version 3
*E. coli*: *Escherichia coli*
EGPA: eosinophilic granulomatosis with polyangiitis
ELISA: enzyme-linked immunosorbent assay
GeoMFI: geometric mean fluorescence intensity
GPA: granulomatosis with polyangiitis
IVD: in vitro diagnostic
MPA: microscopic polyangiitis
MPO: myeloperoxidase
NETs: neutrophil extracellular traps
NAPDH: nicotinamide adenine dinucleotide phosphate-oxidase
PMA: phorbol 12-myristate 13-acetate
PMN: polymorphonuclear leukocytes
PR3: proteinase 3
ROS: reactive oxygen species
SLE: systemic lupus erythematosus
SLEDAI-2 K: Systemic Lupus Erythematosus Disease Activity Index 2000
SLICC/ACR-DI: Systemic Lupus International Collaborative Clinics/American College of Rheumatology damage index
