# Supplementary material for: Impaired phagocytosis and reactive oxygen species production in phagocytes is associated with systemic vasculitis
Source: Arthritis Res Ther. 2016 Apr 22;18:92. doi: 10.1186/s13075-016-0994-1 (PMC4840900; doi:10.1186/s13075-016-0994-1)

A, Gating strategies to evalate the expression of surface markers


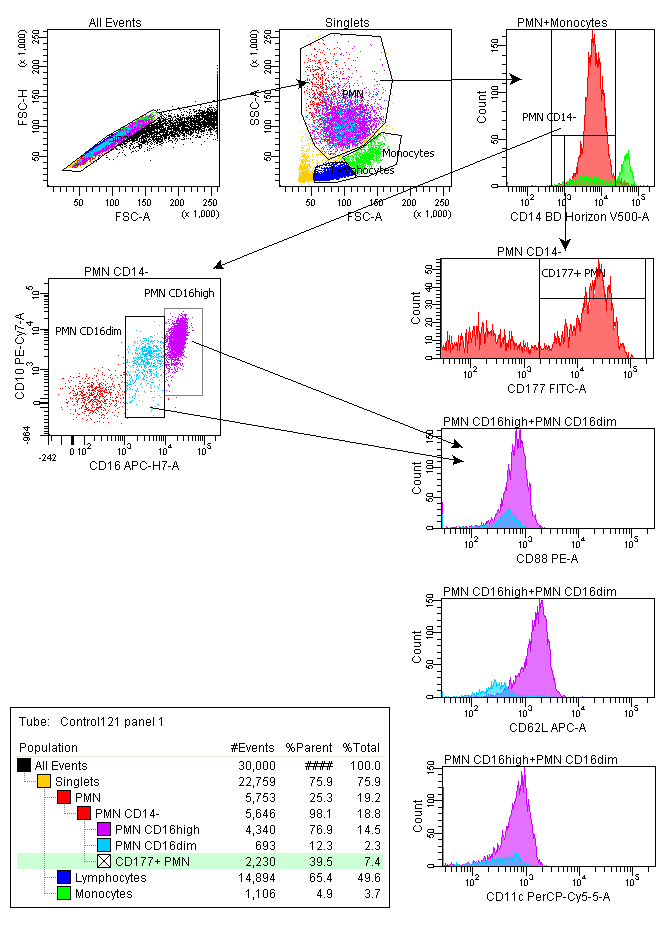


B, Gating strategies to evaluate phagocytosis
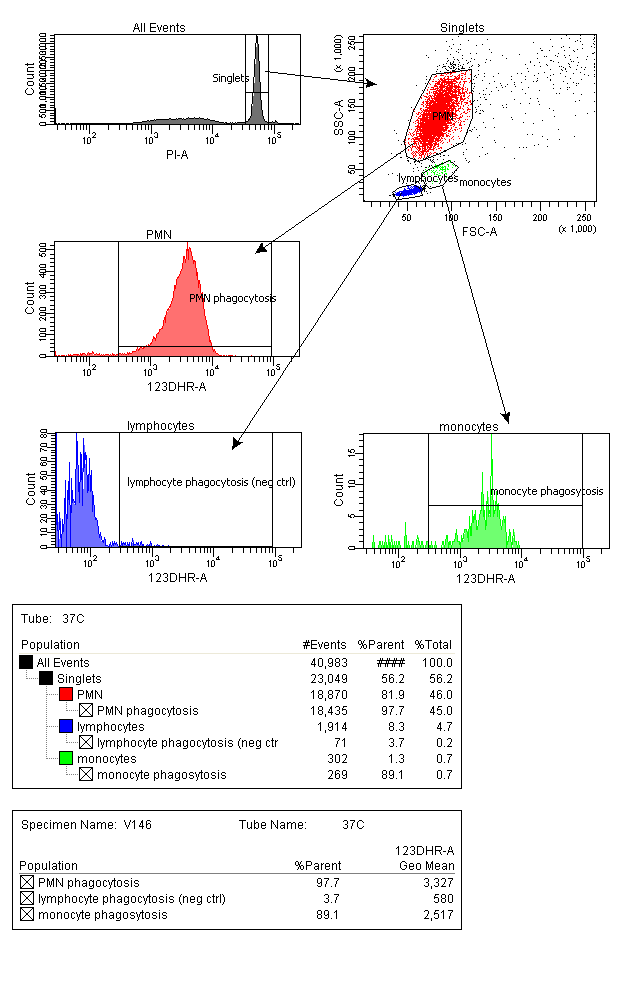


B, Gating strategies to evaluate production reactiv oxygen species.


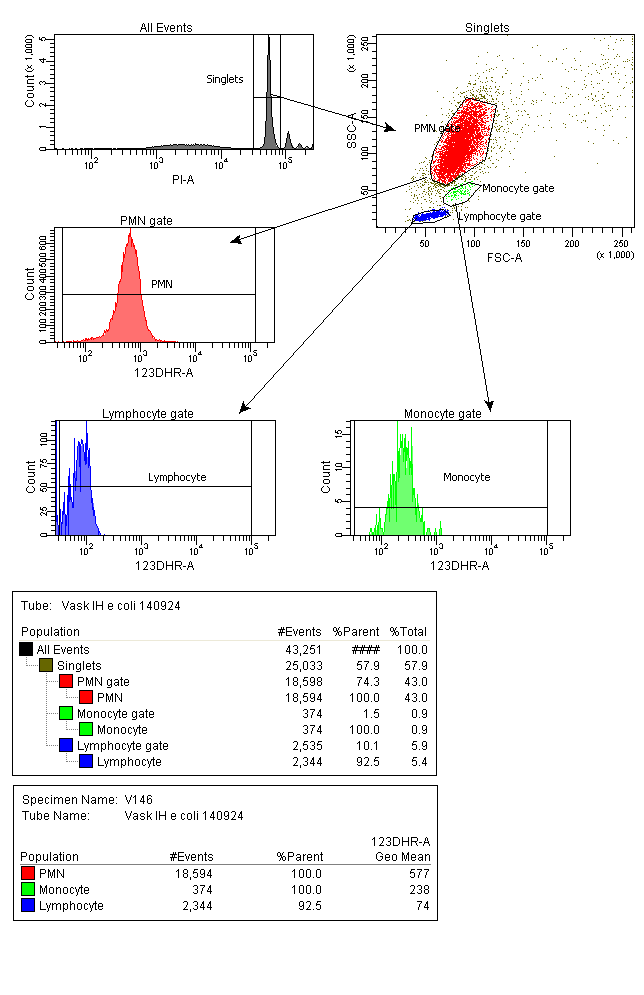

Supplement: Additional file 1: — Gating strategies. (DOCX 188 kb) [file 13075_2016_994_MOESM1_ESM.docx]
